# Hospital Festivals and Meetings

**Published:** 1894-02-10

**Authors:** 


### HOSPITAL FESTIVALS AND MEETINGS.

### GENERAL LYING-IN HOSPITAL, YORK ROAD, S.E.

The annual meeting of the Governors of the General Lying-in Hospital was held on January 31st, with Mr. Henry Owen (in the unavoidable absence of the Earl of Darnley) in the chair; telegrams regretting absence were received from Lord Saye and Sele and Mr. Bousfield. The report for the year chronicled an excess of expenditure over income of £242 16s. 11½d. (in spite of the strict economy exercised by the management) owing to an increase in the number of patients—larger than in any previous year—and to various necessary improvements carried out in sanitary arrangements, accommodation, &c. The medical report stated that during the past year the hospital had been quite free from puerperal fever; of 527 in-patients, two only had died, one from consumption, the other from convulsions arising from disease of the kidneys; and of the 535 children born in the hospital, 496 had left it alive and well. The number of out-patients was 1,527, of whom five had died. It was further stated that all the midwives trained at the hospital that year had passed the necessary examination, with the exception of six who were about to enter for it. Among those present were the Hon. and Rev. Canon Pelham, Dr. Champneys, and Dr. Boxall, who spoke warmly in praise of the untiring services of the medical officers. The chairman, in conclusion, congratulated the committee on the marked improvement in efficiency and economy of the management of the hospital.

### THE FRENCH HOSPITAL.

The annual festival dinner in aid of the funds of the French Hospital and Dispensary, Shaftesbury Avenue, was held at the Hotel Metropole on Saturday last, and was well attended, the chair being taken by the new French Ambassador, M. Decrais. The Lord Mayor was unable, through illness, to be present, and was represented by Sir Polydore de Keyser. Amongst others present were the Italian Ambassador, Count Tornielli, the Swiss Chargé d'Affairs, M. Bourcart, Captain Le Clerc, Comte Du Pentavice de Heussey, Comte de St. Genys, M. J. Knect, M. C. Tailleper, M. Joostens, M. Mimaux, Sir William MacCormac, and Mr. Ernest Rüffer.

The French Hospital has been in existence some twenty-seven years, and was enlarged in 1878. It succours many patients of all nationalities every year, and at present is urgently in need of increased funds to efficiently carry on its work. The speeches at Saturday's banquet left the affairs of the hospital somewhat in the background, being largely complimentary in character. The serious loss sustained by the French colony in London by the death of M. Waddington was referred to by the Chairman, who spoke warmly of the esteem and honour in which his predecessor was universally held, and of the interest he had always taken in the hospital. Sir William MacCormac, in responding to the toast of "The Medical Staff," commended the hospital as "a model of good management." At the end of the evening a short report of its financial condition was read by the Hon. Treasurer, and, as the result, donations to the amount of £2,500 were announced.

